# Complications of Catheter Ablation for Atrial Fibrillation in Patients with Rheumatic Diseases

**DOI:** 10.3390/jcm15093478

**Published:** 2026-05-01

**Authors:** Jenna J. Port, Ariel Furer, Kathleen L. Oakes, Lauren C. Ehrhardt-Humbert, Kevin J. John, Jennifer Chee, Margaret Infeld, Munther K. Homoud, Christopher A. Madias, Guy Rozen

**Affiliations:** 1 Department of Medicine, Tufts Medical Center, 800 Washington St, Boston, MA 02111, USA; jport@tuftsmedicalcenter.org (J.J.P.); kathleen.oakes@tuftsmedicine.org (K.L.O.);; 2 Tufts Medical Center, Cardiac Arrhythmia Center, Cardiovascular Institute, 800 Washington St, Boston, MA 02111, USAjennifer.chee@tuftsmedicine.org (J.C.); margaret.infeld@tuftsmedicine.org (M.I.); munther.homoud@tuftsmedicine.org (M.K.H.); christopher.madias@tuftsmedicine.org (C.A.M.); 3 Faculty of Medicine, The Hebrew University of Jerusalem, Ein Kerem, Jerusalem 9112102, Israel; 4 School of Medicine, Tufts University, Boston, MA 02111, USA

**Keywords:** atrial fibrillation, catheter ablation, autoimmune disease, rheumatic disease, complications

## Abstract

**Background**: Rheumatic diseases (RDs) are associated with increased cardiovascular morbidity, including a 40% higher risk of atrial fibrillation (AF). While ablation has become the cornerstone of rhythm control, its safety in patients with rheumatic diseases remains poorly defined. **Methods**: Adults with a primary admission diagnosis of AF catheter ablation from 2016 to 2022 were identified using the National Inpatient Sample. We excluded patients with other forms of supraventricular tachycardia, pacemaker/defibrillator procedures, and atrioventricular junction ablations. Sociodemographic, clinical characteristics, and outcomes were compared between groups. Multivariate logistic regression adjusted for age, race, sex, and potential comorbid confounders was used to assess for independent associations. **Results**: A weighted total of 48,855 patients were included, 2.5% of which had RD. These patients were predominantly female, older, and had higher rates of renal dysfunction, hypertension, heart failure, history of stroke, ischemic heart disease, heart failure, and obstructive sleep apnea (all *p* < 0.001). Patients with RD had higher complication rates (12.9% vs. 8.8%, *p* < 0.001); specifically, bleeding (*p* < 0.001), infection (*p* = 0.008), pericardial (*p* = 0.003), and respiratory complications (*p* < 0.001). RDs were not found to be an independent predictor of complications, though there was a trend towards more complications (odds ratio 1.43, 95% confidence interval 0.97–2.11, *p* = 0.070). **Conclusions**: Patients with RD undergoing AF ablation were older, female, and had higher rates of comorbidities. This translated to higher unadjusted periprocedural complications in patients with rheumatic diseases. While RDs were not independently associated with adverse outcomes, a trend towards increased complications was observed.

## 1. Introduction

Rheumatic diseases (RDs) are systemic inflammatory conditions that often have extra-articular manifestations, particularly related to the cardiovascular system. Patients with rheumatic diseases are 50% more likely to die from cardiovascular causes than the general population and are particularly vulnerable to rhythm and conduction abnormalities [1,2]. A recent nationwide study reported that patients with RD have up to a 40% increased risk to develop atrial fibrillation (AF) and up to a 1.4 times higher likelihood of hospitalization for AF [3,4,5,6,7,8,9].

Amongst patients with rheumatic diseases who develop AF, the complications can cause significant morbidity and mortality. Patients with RD and AF have higher risk of all-cause death, thrombotic events (ischemic stroke, acute myocardial infarction, pulmonary embolism), and higher rates of hemorrhagic events (such as intracranial and gastrointestinal bleeding) [10]. AF in RD is also linked to higher rates of heart failure and coronary artery disease, compared to AF in the general population [11]. While the pathophysiology of the relationship between AF and RD is poorly understood, increased systemic inflammation is hypothesized to be the driver of AF occurrence and thromboembolic events [12].

Given the burden of cardiovascular disease in this population, and particularly the risks associated with untreated AF, establishing safe strategies for preventing AF recurrences in this population is paramount. Catheter ablation (CA) for atrial fibrillation has become increasingly utilized given its superiority in rhythm and symptom control when compared to antiarrhythmic drugs [13,14,15,16,17]. The data of catheter ablation in patients with rheumatic disease remain limited, and are largely derived from small, single-center cohorts or have not included enough patients to provide a comparison of the procedural complications between patients with and without RD [12,18,19]. These studies have provided important mechanistic and efficacy insights, but have offered limited characterization of peri-procedural risk.

To the best of our knowledge, no large-scale, population-based studies have specifically evaluated the periprocedural safety and complication rates of catheter ablation in patients with RD. We therefore sought to analyze the safety of AF catheter ablation using a nationwide, real-world dataset of patients with and without rheumatic diseases while accounting for differences in baseline comorbidity burden.

## 2. Materials and Methods

### 2.1. Data Source

Our data were sourced from the National Inpatient Sample, the largest publicly available collection of all-payer inpatient data for hospitalizations in the United States of America (USA) (Data S1) [20]. The database contains both patient-level and hospital-level factors; these include patient demographics, primary and secondary hospitalization diagnoses, procedure codes and timing, length of stay, mortality, as well as hospital bed size, region, and teaching status. For this study, we obtained data for the years 2016 to 2022.

### 2.2. Study Population and Variables

**Figure 1:**
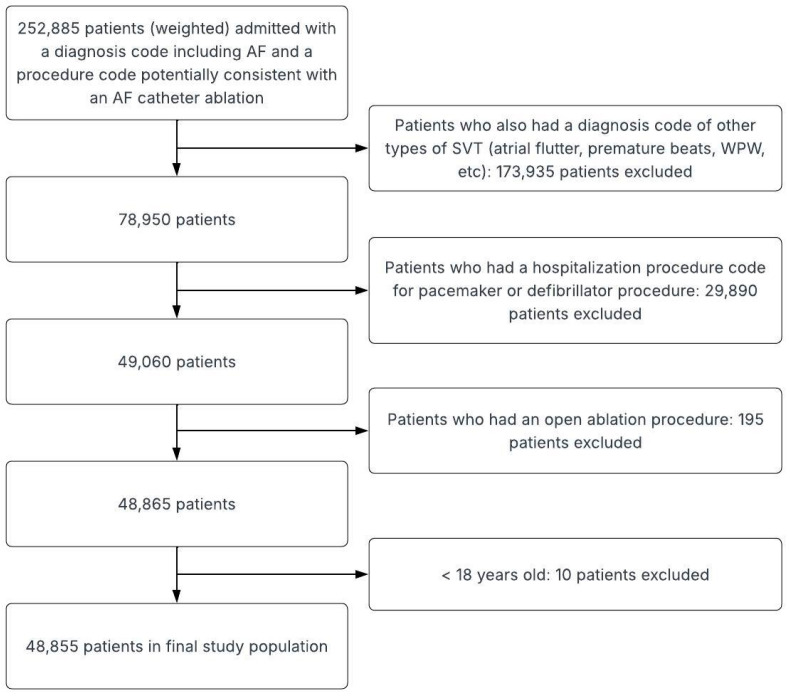
Flow diagram of study population selection.

We collected NIS-reported basic demographic data (age, sex, race) to assess differences in the baseline characteristics for our population of interest. For our study period, the diagnosis and procedure codes used the International Classification of Diseases, Tenth Revision, Clinical Modification (ICD-10-CM). Our target population was patients aged 18 years or older who had a diagnosis code of AF, as well as a procedure code for a CA (Table S1). To identify only those with AF, we excluded patients that have a diagnosis of atrial flutter, pre-excitation syndromes, premature beats, paroxysmal tachycardia as well as those who have the codes for the presence of a pacemaker or defibrillator. To avoid the inclusion of patients who underwent a procedure for ablation of the atrioventricular junction, we excluded patients with diagnostic or procedural codes for defibrillator and pacemaker placements or adjustments. Additionally, we excluded any open surgical procedure (Figure 1, Table S1). This methodology has been used in several studies to isolate only patients with AF undergoing catheter ablation from NIS databases [14,21,22].

To identify patients with rheumatic disorders, we included those with rheumatoid arthritis, systemic lupus erythematous, scleroderma, ankylosing spondylosis, as well as vasculitis and other systemic connective tissue disorders (Table S2).

### 2.3. Study Outcomes

Our primary study outcomes included the known complications for an AF catheter ablation, as described in the AF ablation literature [14,21]: pericardial (hemopericardium, tamponade, need for pericardiocentesis, and pericarditis), cardiac (cardiac arrest, arrythmia, myocardial infarction, congestive heart failure), pulmonary (pneumothorax, hemothorax, respiratory failure, diaphragm paralysis, other respiratory compromise), intra-op or post-procedural hemorrhage or hematoma, vascular (complication requiring surgical repair, accidental puncture/laceration, arteriovenous fistula), infection (including bacteremia and sepsis), and neurologic (cerebrovascular accident, transient ischemic attack). To avoid overlapping classifications, ICD codes were assigned uniquely to a single category; when a code could potentially fall under multiple organ systems, it was categorized according to the organ system primarily affected. These codes were selected based on a review of the pertinent literature and matched with other studies to maintain consistency [13,14,21,22,23]. Please see Table S3 for the complete list of the ICD-10-CM codes used to identify complication rates. The secondary outcome was in-hospital all-cause mortality. Length of stay was also compared between groups as a utilization metric.

### 2.4. Statistical Analysis

All statistical analysis was performed on weighted data, as described in Data S1. Missing data was not included in the final analysis. Descriptive statistics were presented as frequencies and percentages for categorical variables and as means for continuous variables. Categorical comparisons were performed using Pearson χ^2^ or Fisher Exact tests while student *t*-tests were used to compare continuous variables. Multivariate logistic regression was used to identify independent predictors of complications. The regression model included age, race, sex, and relevant comorbidities (diabetes, renal dysfunction, hypertension, heart failure, history of stroke, ischemic heart disease, obesity, and obstructive sleep apnea).

Data analysis and visualization were performed using Python (v3.12.7, Python Software Foundation)in Jupyter Notebook (v7.2.2). The pandas (v2.2.1) library was used for data manipulation, while NumPy (v1.26.4), SciPy (v1.12.0), and statsmodels.api (v0.14.2) were used for statistical analysis. Data visualization was performed with matplotlib (v3.8.3). A *p*-value of <0.050 was considered statistically significant.

## 3. Results

### 3.1. Baseline Characteristics

A total of 9771 unweighted hospitalizations between January 2016 and December 2022 were included in the analysis, representing 48,855 weighted hospitalizations during which CA for AF was performed. Within this weighted cohort, 2.5% (1205) patients had a diagnosis of RD. The median age of patients without RD was 67 (interquartile range [IQR] 61–73) and was 70 (IQR 63–77) for those with RD (*p* < 0.001). Patients with rheumatic diseases were more likely to be female (66.4% versus 39.6%, *p* < 0.001), black (7.5% versus 5.0%, *p* < 0.001) and have higher rates of comorbidities, particularly renal dysfunction (18.7% versus 14.7%, *p* < 0.001), hypertension (81.3% versus 76.8%, *p* < 0.001), heart failure (39.0% versus 33.3%, *p* < 0.001), ischemic heart disease (24.1% versus 17.1%, *p* < 0.001), and obstructive sleep apnea (31.5% versus 25.2%, *p* < 0.001). Additionally, RD patients were more likely to be insured under Medicare (72.2% versus 58.1%, *p* < 0.001). Complete demographic and clinical characteristics of the study population are presented in Table 1.

### 3.2. Overall Rates of In-Hospital Complications

**Figure 2:**
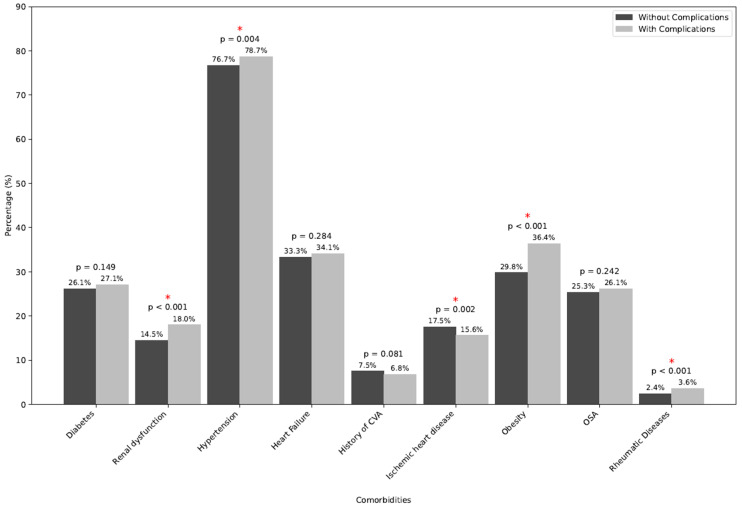
Frequencies of comorbid conditions in patients with and without procedural complications. The *p*-values refer to changes in frequencies of selected comorbidities in patients with and without complications. They were calculated using a chi-squared test, and the significant values were denotated with an asterisk.

When analyzing the entire population, at least one complication occurred in 8.93% (*n* = 4365) of cases during the study period (Table 2) and all-cause in-hospital mortality was documented in 0.79% (*n* = 385) of cases. Patients who experienced complications were more likely to have concurrent comorbidities; in particular, renal dysfunction (*p* < 0.001), hypertension (*p* = 0.004), obesity (*p* < 0.001), and/or RD (*p* < 0.001) (Figure 2).

### 3.3. Complications in Patients with Rheumatic Diseases

Patients with a diagnosis code of RD had significantly higher rates of immediate peri-procedural complication following an AF catheter ablation (12.9% versus 8.8%, *p* < 0.001) (Table 2). This increased association with complications is driven by significantly higher rates of hemorrhagic (3.7% versus 1.3%, *p* < 0.001), infectious (1.2% versus 0.6%, *p* = 0.008), pericardial (5.4% versus 3.7%, *p* = 0.003), and respiratory (2.9% versus 1.5%, *p* < 0.001) complications (Table 2). These patients also had on average longer hospitalizations (mean stay of 4.08 days versus 3.69 days, *p* = 0.001), (Table 2). There was no evidence that the atrial fibrillation subtype (paroxysmal compared to persistent) increased the association between rheumatic disease and procedural complications (*p* = 0.730). There were no deaths in patients with RD who underwent CA during this study period.

Analysis of specific rheumatic disease subgroups revealed variations in complication rates and hospitalization durations. In patients with rheumatoid arthritis (RA), the rate of complications was significantly higher compared to those without RA (13.38% versus 8.86%, *p* < 0.001) and these patients also experienced longer hospitalizations on average (4.01 days versus 3.69 days, *p* = 0.023). Similarly, patients with scleroderma had higher rates of complications (25.00% versus 8.92%, *p* < 0.001), though these patients did not experience statistically longer hospital stays (*p* = 0.952). No significant differences were observed in the rate of complications, mortality, or hospitalization lengths among patients with systemic lupus erythematosus (SLE), ankylosing spondylosis, or other non-specific connective tissue diseases although these groups comprised relatively few patients.

### 3.4. Predictors of Complications and Mortality

The multivariate analysis for independent associations with higher risks of in-hospital complications is presented in Table 3. Female sex (odds ratio (OR) [confidence interval (CI)]: 1.25 [1.08–1.45], *p* = 0.002), white race (1.27 [1.04–1.54], *p* = 0.018), obesity (1.36 [1.16–1.59], *p* < 0.001), and renal dysfunction (1.32 [1.08–1.61], *p* = 0.006) were found to be independently associated with increased complication rates. While a diagnosis code of RD was not found to be an independent predictor of AF complications, a trend towards increased procedural complications was present (1.43 [0.97–2.11], *p* = 0.070) (Table 3). Sub-analysis of paroxysmal or persistent AF did not increase the strength of the association (1.37 [0.77–2.46], *p* = 0.283 and 1.39 [0.77–2.52], *p* = 0.279 respectively). When adjusted for the same comorbidities, neither RA (1.51 [0.95–2.42], *p* = 0.083), scleroderma (3.40 [0.68–16.94], *p* = 0.136), nor SLE (1.28 [0.45–3.68], *p* = 0.643) were found to be independently associated (Table S4).

## 4. Discussion

To our knowledge, this is the largest, population-based study specifically evaluating periprocedural complications of AF CA in patients with rheumatic diseases compared to general population. Using data from the NIS database, the largest inpatient dataset of hospitalizations in the US, we identified a weighted total of 1205 patients with RD who underwent an AF catheter ablation. These patients were predominantly female and had higher rates of cardiovascular comorbidities (hypertension, heart failure, ischemic heart disease), obstructive sleep apnea, renal dysfunction, and a history of stroke. Additionally, we observed a significant difference in primary payer distribution, with RD patients more frequently covered by Medicare compared to those without RD.

Although patients with diagnosis codes of rheumatic diseases were found to have a significantly higher rate of complications compared to those without, this association did not reach statistical significance in a multivariate analysis (*p* = 0.07), implying that the difference might be driven by the higher rate of baseline comorbidities and the female sex of these patients. This trend towards higher risk of peri-procedural complications in RD patients needs to be further investigated prospectively to answer this question definitively.

Prior studies have typically examined individual rheumatic disease, such as RA, or have focused on late outcomes, such as AF recurrence following ablation, and not the periprocedural complication rates themselves [12,18,19]. These analyses have been limited by small sample sizes, reducing the power to detect relatively infrequent procedural complications. In contrast, the NIS captures a large inpatient population with higher baseline acuity, enabling more robust evaluation of rare adverse events [23,24]. This characteristic enables improved analysis of infrequent complications, as demonstrated in this paper. We were able to analyze both the overall complication rates and identify the specific subtypes more common in patients with RD, including hemorrhagic, infectious, pericardial, and respiratory complications.

Hemorrhagic complications showed the most significant difference when comparing patients with RD to the non-RD population. There have been multiple studies that have shown increased bleeding events in patients with RD, attributed to increased use of glucocorticoids, NSAIDs, small vessel vasculopathy and systemic inflammation [10,25,26]. These patients may additionally have a higher risk of developing either autoantibodies against coagulation factors or immune thrombocytopenia [27,28]. The nearly two-fold increased infection risk in post-CA RD patients could similarly be explained by the immunosuppression and glucocorticoids typically used to control disease activity [29]. The increased rate of pericardial complications (including pericarditis, hemopericardium, and tamponade) could be explained by the coagulopathy as explained previously, in addition to the baseline increased systemic inflammation and immune dysregulation decreasing the threshold for the development of myocardial and pericardial inflammation following an ablation [30,31,32]. The pathophysiologic connection between increased rates of respiratory complications and AF catheter ablation is a little less clear, though may be due to the high rate of pre-existing pulmonary disease in patients with RD (30–40% in RA, nearly 50% in systemic sclerosis, and 41% in patients with inflammatory myopathies) [33,34,35,36,37].

While these mechanisms offer compelling pathophysiological explanations for the observed increase in unadjusted complication rates with those who have RD, multivariate analysis tempered this observation. When adjusted for comorbidities frequently seen in AF (chronic kidney disease, obesity, hypertension, diabetes, and OSA), the association between RD and procedural complications was attenuated, though there was a trend towards higher adjusted complication rates. These comorbid conditions are often more prevalent in patients with RD, driven both by the underlying systemic inflammation of these diseases and the effects of medications like glucocorticoids and immunomodulators [38,39,40,41]. Our findings suggest that it is not solely the systemic inflammatory conditions themselves, but rather the associated comorbidities that largely account for the increased complication rates observed in this population.

However, the *p*-value of 0.070 does suggest a residual effect that warrants further investigation. The current body of evidence regarding the safety and efficacy of catheter ablation for atrial fibrillation has shown that patients with underlying rheumatic diseases tend to have comparable long-term outcomes, though they do have a higher risk of early recurrence [19,42]. Taken together with our findings of borderline statistical significance for increased procedural complications, these data suggest rheumatic disease alone should not be considered a contraindication for ablation, but rather a marker of higher baseline risk that warrants careful patient selection and periprocedural optimization.

Given the high prevalence of heart failure and cardiometabolic comorbidities in patients with RD, optimization of guideline-directed medical therapy represents an important parallel strategy while patients are under evaluation for procedural intervention. In particular, sodium-glucose cotransporter-2 inhibitors (SGLT2i) have emerged as a foundational therapy in heart failure, and have also been seen to have pleiotropic benefits, including possible antiarrhythmic effects [43]. Emerging data suggests that these therapies may be especially relevant in populations with heightened inflammatory and cardiometabolic risk, such as those with rheumatic diseases. Contemporary heart failure and cardiometabolic therapies may present an important avenue to both reduce AF burden and potentially mitigate procedural risk in this population.

Exploring emerging procedural strategies that may mitigate risks in this unique population presents a potential area for future research. This study has noted specific increased hemorrhagic, pericardial, and infectious complications in the RD population. Pulsed Field Ablation (PFA) is newer technology that may provide unique advantages in those with RD, specifically relating to its lower risk of pericardial complications given the less traumatic design of PFA catheters and no risk of steam pop, as well as decreased risk of pulmonary vein stenosis and pulmonary hypertension, particularly in this population that may already have pulmonary vascular involvement [44,45].

Although this study was not intended to assess long-term clinical outcomes, the available literature suggests that patients with rheumatic diseases may derive significant net benefit from CA, despite the potentially higher procedural risk. Patients with autoimmune rheumatic diseases and atrial fibrillation have substantially elevated baseline risks of stroke, heart failure, and mortality compared to non-RD populations, even when treated with contemporary medical therapy [1,2,3,4,5,6,7,8,9,10,11]. In this context, improvements in rhythm control presumably translates into greater absolute risk reduction. Catheter ablation, when coupled with careful medical optimization of comorbidities, offers clinically meaningful benefit in appropriately selected patients.

### Limitations

Our study utilizes the NIS, a large and widely used database designed to capture administrative data on hospital discharges across the United States. While the NIS is a valuable and generalizable resource, it is not without limitations. As an administrative database originally designed for cost and utilization, the NIS is not optimized for causal inference and therefore observed associations cannot be interpreted as causal effects. Since the database relies on administrative reports from individual hospitals, it is subject to misclassification bias of AF ablations and RD diagnoses. Given the absence of a single ICD-10 code specific to atrial fibrillation catheter ablation, we applied stringent exclusion criteria to better isolate this population; however, this approach may introduce selection bias by excluding more complex cases, potentially limiting generalizability to higher-risk or more advanced procedures. The difference in primary payer between those with RD and those without may reflect differences in socioeconomic status and age distribution, and could introduce treatment-related confounding related to site of care, resource utilization, and discharge practices. While this variable may influence variables such as hospitalization cost and length of stay, it is less likely to account for differences in periprocedural complication rates, which are more directly related to patient-level clinical risk.

As the analysis is limited to diagnoses listed in the ICD-10 database, granular detail regarding AF ablation modality, specific anticoagulation medication, and rheumatic disease activity cannot currently be assessed. Since the NIS only includes inpatient hospitalizations, it is inherently biased towards overall higher-risk patients, as lower-risk outpatient procedures are not represented. While there is a selection bias toward higher-risk patients, the comparisons within this study should remain internally valid, as this study compares like-to-like. Consequently, the observed associations are expected to hold, even though the absolute complication rates are higher than would be seen in broader contemporary practice. Lastly, because the NIS represents hospitalization events rather than unique patients, repeated admissions for the same individual cannot be distinguished, potentially leading to overrepresentation of complications among patients with severe or recurrent disease, a scenario that may be more common in patients with rheumatic diseases.

Despite these limitations, the NIS remains one of the largest and most comprehensive sources of inpatient data, allowing for robust population-level analysis. Our findings provide valuable insights that can inform clinical decision-making, though additional studies incorporating outpatient data are warranted to validate these results and further explore the mechanisms underlying our observations.

## 5. Conclusions

This is the largest analysis to date of complications associated with catheter ablations for atrial fibrillation in patients with rheumatic diseases. Compared to those without RD, patients with RD undergoing AF ablation during the study period were older, more likely to be female and/or black, and had a greater burden of comorbidities. This translated to higher periprocedural complications and longer lengths of stay in patients with RD. While multivariable analysis did not reach statistical significance, the observed trend suggests that RD may still be clinically relevant, and further studies are needed to better assess this association.

## Figures and Tables

**Table 1:** Baseline characteristics of the study population. The *p*-values were calculated using the chi-squared test to analyze changes in frequency in patients with and without specified rheumatic diseases.

	Overall	Without Rheumatic Diseases	With Rheumatic Diseases	*p*-Value
Unweighted count, *n*	9771	9530	241	
Weighted count, *n*	48,855	47,650	1205	
Age				
18–34 y	0.8% (385)	0.8% (380)	0.4% (5)	0.187
35–49 y	5.9% (2885)	6.0% (2865)	1.7% (20)	<0.001
50–64 y	32.2% (15,710)	32.4% (15,420)	24.1% (290)	<0.001
65–79 y	53.2% (25,985)	52.9% (25,210)	64.3% (775)	<0.001
>80 y	8.0% (3890)	7.9% (3775)	9.5% (115)	0.046
Sex				
Male	59.8% (29,205)	60.4% (28,800)	33.6% (405)	<0.001
Female	40.2% (19,650)	39.6% (18,850)	66.4% (800)	<0.001
Race				
White	81.9% (40,005)	81.9% (39,010)	82.6% (995)	0.556
Black	5.1% (2485)	5.0% (2395)	7.5% (90)	<0.001
Hispanic	5.8% (2820)	5.8% (2755)	5.4% (65)	0.612
Asian or Pacific Islander	1.8% (855)	1.8% (835)	1.7% (20)	0.896
Native American	0.3% (160)	0.3% (160)	0.0% (0)	0.078
Payment type				
Medicare	58.5% (28,565)	58.1% (27,695)	72.2% (870)	<0.001
Medicaid	5.4% (2655)	5.5% (2615)	3.3% (40)	0.001
Private Insurance	32.4% (15,825)	32.6% (15,550)	22.8% (275)	<0.001
Self-pay	1.0% (500)	1.0% (500)	0.0% (0)	<0.001
No Charge	0.1% (60)	0.1% (60)	0.0% (0)	0.414
Hospital Region				
Northeast	19.5% (9505)	19.6% (9340)	13.7% (165)	<0.001
Midwest	11.9% (5835)	11.9% (5680)	12.9% (155)	0.341
South	30.8% (15,070)	30.8% (14,660)	34.0% (410)	0.017
West	9.7% (4745)	9.7% (4635)	9.1% (110)	0.520
Comorbidities				
Diabetes	26.2% (12,815)	26.2% (12,470)	28.6% (345)	0.055
Renal Dysfunction	14.8% (7245)	14.7% (7020)	18.7% (225)	<0.001
Hypertension	76.9% (37,580)	76.8% (36,600)	81.3% (980)	<0.001
Heart Failure	33.4% (16,320)	33.3% (15,850)	39.0% (470)	<0.001
History of CVA	7.4% (3625)	7.3% (3495)	10.8% (130)	<0.001
Ischemic heart disease	17.3% (8450)	17.1% (8160)	24.1% (290)	<0.001
Obesity	30.4% (14,850)	30.4% (14,495)	29.5% (355)	0.475
OSA	25.4% (12,400)	25.2% (12,020)	31.5% (380)	<0.001

**Table 2:** Complication rates among patients with and without rheumatic diseases. *p*-values refer to changes in frequency of the specified complication in patients with and without rheumatic diseases. The *p*-value for length of stay was calculated using a two-sample *t*-test. For all other variables, the chi-squared test was used.

Complication	Overall	Without Rheumatic Diseases	With Rheumatic Diseases	*p*-Value
Cardiac, % (*n*)	1.65% (805)	1.7% (790)	1.2% (15)	0.266
Hemorrhage/Hematoma, % (*n*)	1.32% (645)	1.3% (600)	3.7% (45)	<0.001
Infection, % (*n*)	0.64% (315)	0.6% (300)	1.2% (15)	0.008
Neurologic, % (*n*)	0.37% (180)	0.4% (180)	0.0% (0)	0.033
Pericardial, % (*n*)	3.78% (1845)	3.7% (1780)	5.4% (65)	0.003
Respiratory, % (*n*)	1.54% (750)	1.5% (715)	2.9% (35)	<0.001
Vascular, % (*n*)	1.80% (880)	1.8% (855)	2.1% (25)	0.470
At least one complication, % (*n*)	8.93% (4365)	8.8% (4210)	12.9% (155)	<0.001
Length of stay, days (SD)	3.70 ± 5.47	3.69 ± 5.50	4.08 ± 4.04	0.001

**Table 3:** Multivariate logistical regression of predictors for procedural complications. A binary logistic regression model was fitted using maximum likelihood estimation. The dependent variable was the presence of any procedural complication. Independent variables included sex, race, age, and comorbidities as listed. Odds ratios (ORs) and 95% confidence intervals (CIs) are reported, with the corresponding *p*-values.

	OR	95% CI	*p*-Value
Sex			
Male	Reference		
Female	1.25	1.08–1.45	0.002
Race			
Non-White	Reference		
White	1.27	1.04–1.54	0.018
Age			
18–34 y	Reference		
35–49 y	0.86	0.35–2.11	0.747
50–64 y	0.96	0.41–2.25	0.923
65–79 y	1.03	0.44–2.41	0.951
>80 y	1.07	0.44–2.58	0.886
Comorbidities ^1^			
Rheumatic Diseases	1.43	0.97–2.11	0.070
Diabetes	0.99	0.84–1.17	0.932
Heart Failure	0.95	0.81–1.11	0.513
History of CVA	0.87	0.66–1.15	0.338
Hypertension	1.04	0.87–1.25	0.661
Ischemic heart disease	0.85	0.70–1.04	0.110
OSA	0.96	0.81–1.14	0.645
Obesity	1.36	1.16–1.59	>0.001
Renal dysfunction	1.32	1.08–1.61	0.006

^1^ Reference values for this section are those without the condition of interest. CVA: cerebrovascular accident; OSA: obstructive sleep apnea.

## Data Availability

The National Inpatient Sample (NIS) dataset used for this study, statistical methods, study material, and analytical tools used for this study will not be made available to other researchers. This is due to the restrictions on the sharing of data in the Healthcare Cost and Utilization Project Data Use Agreement. However, the database is publicly available for purchase and the detailed materials and methods described in this paper will make it possible for anyone to replicate this study and reproduce our results.

## Supplementary Materials

The following supporting information can be downloaded at https://www.mdpi.com/article/10.3390/jcm15093478/s1, Data S1: Supplemental Data; Table S1: Inclusion and exclusion codes; Table S2: Comorbidities and rheumatic disease codes; Table S3: Complication codes; Table S4: Multivariate analysis for those with rheumatoid arthritis, scleroderma, and systemic lupus erythematosus (SLE).

## Abbreviations

The following abbreviations are used in this manuscript:

AF	Atrial Fibrillation
CA	Catheter Ablation
ICD-10-CM	International Classification of Diseases, Tenth Revision, Clinical Modification
NIS	National Inpatient Sample
RA	Rheumatoid Arthritis
RD	Rheumatic Disease
SLE	Systemic Lupus Erythematosus
USA	United States of America
