# Supplementary material for: Complications of Catheter Ablation for Atrial Fibrillation in Patients with Rheumatic Diseases
Source: J Clin Med. 2026 May 1;15(9):3478. doi: 10.3390/jcm15093478 (PMC13163545; doi:10.3390/jcm15093478)
Supplement: Supplementary file 1 [file jcm-15-03478-s001.zip › RD_AFibAblation_SuppData.pdf]

<u>Supplemental Materials</u>

Data S1:

Supplemental Data: The National Inpatient Sample (NIS) is a nationwide database developed for the Healthcare Cost and Utilization Project (HCUP). For each index hospitalization, the database includes either 30 diagnosis codes and 15 procedure codes (in 2016) or 40 diagnosis codes and 25 procedure codes (from 2017-2020)**.** Unweighted, it provides data on 7 million hospitalizations per year, amounting to approximately 20% stratified samples of inpatient discharges from US hospitals (47 states in total), which covers more than 97% of the US inpatient population. This data can be used to calculate national estimates by using the sampling weights provided by the NIS. The nationwide estimates of the entire US population are calculated using a standardized sampling and weighting method provided by HCUP.

Table S1: Inclusion and Exclusion Codes

Inclusion Codes	
Atrial fibrillation codes	I480, I4891, I482, I4819, I481, I489, I480, I4811, I4821, I48, I4820
Catheter ablation codes	025S3ZZ, 025T3ZZ, 02573ZZ, 02583ZZ
**Exclusion Codes**	
Atrial flutter	I483, I484, I4892
Paroxysmal tachycardia	I47x
Premature Beat	I491, I492, I493, I494, I4940, I4949, I498, I499, I495
Excitable Syndrome	I456, I458x, I4581
Pacemaker Presence	Z950, Z4501x, Z4502
Pacemaker Procedure	0JH604Z, 0JH605Z, 0JH606Z, 02H40JZ, 02H43JZ, 02H43NZ, 02H60NZ, 02H63JZ, 02H70JZ, 02HK0JZ, 02HK0NZ, 02HK3JZ, 02HK3NZ, 02HL0JZ, 02HL0NZ, 02HL3JZ, 02HL3NZ, 02HN4JZ, 02PA0NZ, 02PA3NZ, 02WA0NZ, 02WA3NZ, 02WA4NZ, 02WAXNZ, 02H40NZ, 02H60JZ, 02H63NZ, 02H70NZ, 02H73JZ, 02H73NZ, 02HN0JZ, 02HN3JZ, 0JH604Z, 0JH606Z, 0JH634Z, 0JH636Z, 0JH804Z, 0JH806Z, 0JH834Z, 0JH836Z, 02H44JZ, 02H44NZ, 02H64JZ, 02H64NZ, 02H74JZ, 02H74NZ, 02HK4JZ, 02HK4NZ, 02HL4JZ, 02HL4NZ, 02PA4NZ, 0JH605Z, 0JH607Z, 0JH635Z, 0JH637Z, 0JH805Z, 0JH807Z, 0JH835Z, 0JH837Z, 0JH606Z
Defibrillator	Z95810, Z4502
Defibrillator Procedure	0JH607, 0JH607Z, 0JH608, 0JH608Z, 0JH609, 0JH609Z, 0JH60F, 0JH60FZ, 0JH638, 0JH638Z, 0JH639, 0JH639Z, 02H40KZ, 02H43KZ, 02H60KZ, 02H70KZ, 02HK3KZ, 02HL0KZ, 02HN0KZ, 02HN3KZ, 02HN4KZ, 02H63KZ, 02H73KZ, 02HK0KZ, 02HL3KZ

Table S2: Comorbidity and Rheumatic Disease Codes

Comorbidities	
Diabetes	E08x, E0800, E0801, E0810, E0811, E08618, E08620, E08621, E08622, E08628, E08630, E08638, E08641, E08649, E0865, E0869, E088, E089, E09x, E0900, E0901, E0910, E0911, E09618, E09620, E09621, E09622, E09628, E09630, E09638, E09641, E09649, E0965, E0969, E098, E099, E10x, E1010, E1011, E10618, E10620, E10621, E10622, E10628, E10630, E10638, E10641, E10649, E1065, E1069, E108, E109, E11x, E1100, E1101, E11618, E11620, E11621, E11622, E11628, E11630, E11638, E11641, E11649, E1165, E1169, E118, E119, E13x, E1300, E1301, E1310, E1311, E13618, E13620, E13621, E13622, E13628, E13630, E13638, E13641, E13649, E1365, E1369, E138, E139, R81
Renal	N181, N182, N1830, N1831, N1832, N184, N185, N186, N189, N19, N18, I120, I129, I1310, I1311
Hypertension	I10, I11, I12, I13, I15, I16, I1A
Heart Failure	I50x, I110, I130, I132, I0981, T8622, I502x, I503x, I504x, I5082, I5081x, I501x, I508x, I509x
History of CVA	Z8673x
Ischemic heart disease	I252, I22x, I257, I258, I259, Z9861, Z955, Y822, Z951, I255
Obesity	Z6825, Z6826, Z6827, Z6828, Z6829, Z6830, Z6831, Z6832, Z6833, Z6834, Z6835, Z6836, Z6837, Z6838, Z6839, E6601, Z6841, Z6842, Z6843, Z6844, Z6845, E65, E6609, E661, E668, E669, E65, E6609, E661, E668, E669, E6601, Z6841, Z6842, Z6843, Z6844, Z6845, Z6835, Z6836, Z6837, Z6838, Z6839, Z6830, Z6831, Z6832, Z6833, Z6834, Z6825, Z6826, Z6827, Z6828, Z6829
OSA	G4733
**Rheumatic Diseases:**	
Rheumatoid Arthritis	M05, M060, M062, M063, M064, M068, M069
Enteropathic arthropathies	M07
SLE	M32
Scleroderma	M34
Hypermobility Syndrome	M357
Ankylosing spondylosis	M45x
Other	M30, M31, M33, M351, M352, M353, M354, M355, M356

Table S3: Complication Codes

Complications:	
Pericardial	S260x, I312, I314, 0W9D30Z, 0W9D3ZX, 0W9D3ZZ, 0W9D4ZX, 0W9D4ZZ, I308, I309
Cardiac	I9771x, I9712x, I9719x, I9713x, I9711x, I9789, I97710, I97790
Respiratory	J938x, J939, J942, J95821, J9588, J986 AND G839, J9589
Hemorrhage/ Hematoma	L7602x, L762, L7622x, L763, L7634x, I97, I974, I9741, I97418, I9742, I976, I9761, I9762, I97620, I97621, I97638, I9763
Vascular	L761, L7612, I975x, I770, K661, 03Q30ZZ, 03Q34ZZ, 03Q40ZZ, 03Q43ZZ, 03Q24ZZ, 03Q24ZZ, 03Q23ZZ, 03Q20ZZ, 03Q14ZZ, 02QX4ZZ, 02QX3ZZ, 02QX0ZZ, 02QW4ZZ, 02QW3ZZ, 02QW0ZZ, 02QR4ZZ, 02QR3ZZ, 02QR0ZZ, 02QQ4ZZ, 02QQ3ZZ, 02QQ0ZZ, 02QP4ZZ, 02QP3ZZ, 02QP0ZZ, 03Q44ZZ, 03Q50ZZ, 03Q53ZZ, 03Q54ZZ, 03Q60ZZ, 03Q63ZZ, 03Q64ZZ, 03Q70ZZ, 03Q73ZZ, 03Q74ZZ, 03Q80ZZ, 03Q83ZZ, 03Q84ZZ, 03Q90ZZ, 03Q93ZZ, 03Q94ZZ, 03QD0ZZ, 03QD3ZZ, 03QD4ZZ, 03QF0ZZ, 03QF3ZZ, 03QF4ZZ, 03QA0ZZ, 03QA3ZZ, 03QA4ZZ, 03QB0ZZ, 03QB3ZZ, 03QB4ZZ, 03QC0ZZ, 03QC3ZZ, 03QC4ZZ, 04Q00ZZ, 04Q03ZZ, 04Q04ZZ, 04QD4ZZ, 04QE0ZZ, 04QE3ZZ, 04QE4ZZ, 04QF0ZZ, 04QF3ZZ, 04QF4ZZ, 04QH0ZZ, 04QH3ZZ, 04QH4ZZ, 04QJ0ZZ, 04QJ3ZZ, 04QJ4ZZ, 04QK0ZZ, 04QK3ZZ, 04QC4ZZ, 04QD0ZZ, 04QD3ZZ, 04QK4ZZ, 04QL0ZZ, 04QL3ZZ, 04QL4ZZ, T81719A, T8172XA, T81718A
Infection	T8140XA, R5082, T8144XA, T8149XA, T8112x, T814x, R6520, T8112XA, R7881
Neurologic	I9781, I97811, I9782x

Table S4: Multivariate analysis for those with rheumatoid arthritis, scleroderma, and systemic lupus erythematosus (SLE). The covariates used in this analysis were the same as the ones used in **Table 3.**

	**Multivariate Analysis**		
	**Odds Ratio**	**95% Confidence Interval**	**p-value**
**Rheumatoid Arthritis**	1.51	0.95 - 2.42	0.083
**Scleroderma**	3.4	0.68 - 16.94	0.136
**SLE**	1.28	0.45 - 3.68	0.643
